# Chitosan as a Tool for Sustainable Development: A Mini Review

**DOI:** 10.3390/polym14071475

**Published:** 2022-04-05

**Authors:** Soundouss Maliki, Gaurav Sharma, Amit Kumar, María Moral-Zamorano, Omid Moradi, Juan Baselga, Florian J. Stadler, Alberto García-Peñas

**Affiliations:** 1 Departamento de Ciencia e Ingeniería de Materiales e Ingeniería Química (IAAB), Universidad Carlos III de Madrid, 28911 Leganés, Spain; soundoussa.maliki@gmail.com (S.M.); mamoralz@ing.uc3m.es (M.M.-Z.); jbaselga@ing.uc3m.es (J.B.); 2 International Research Centre of Nanotechnology for Himalayan Sustainability (IRCNHS), Shoolini University, Solan 173212, India; dramitchem@gmail.com; 3 College of Materials Science and Engineering, Shenzhen Key Laboratory of Polymer Science and Technology, Guangdong Research Center for Interfacial Engineering of Functional Materials, Nanshan District Key Laboratory for Biopolymers and Safety Evaluation, Shenzhen University, Shenzhen 518060, China; fjstadler@szu.edu.cn; 4 School of Science and Technology, Glocal University, Saharanpur 247001, India; 5 Department of Chemistry, Shahr-e-Qods Branch, Islamic Azad University, Tehran 61349, Iran; moradi.omid@gmail.com

**Keywords:** chitosan, sustainable development, circular economy, biopolymers

## Abstract

New developments require innovative ecofriendly materials defined by their biocompatibility, biodegradability, and versatility. For that reason, the scientific society is focused on biopolymers such as chitosan, which is the second most abundant in the world after cellulose. These new materials should show good properties in terms of sustainability, circularity, and energy consumption during industrial applications. The idea is to replace traditional raw materials with new ecofriendly materials which contribute to keeping a high production rate but also reducing its environmental impact and the costs. The chitosan shows interesting and unique properties, thus it can be used for different purposes which contributes to the design and development of sustainable novel materials. This helps in promoting sustainability through the use of chitosan and diverse materials based on it. For example, it is a good sustainable alternative for food packaging or it can be used for sustainable agriculture. The chitosan can also reduce the pollution of other industrial processes such as paper production. This mini review collects some of the most important advances for the sustainable use of chitosan for promoting circular economy. Hence, the present review focuses on different aspects of chitosan from its synthesis to multiple applications.

## 1. Introduction: Necessity of Alternative Materials for a Circular Economy

The new regulations promoted by numerous governments are trying to take care of the environment by protecting actions and behaviors to develop a new sustainable economy. Some of the most important goals of these laws are aimed at the reduction of the excessive consumption of non-renewable raw materials, especially those derived from natural sources. The extraction and cleaning of raw materials are responsible for soil degradation, biodiversity loss, water shortages, and global warming. The use of residues as raw materials is a new concept derived from the circular economy which could definitely contribute to the reduction of the huge amounts of trash accumulated in landfills. The concept of a circular material means that a new product can be obtained from the old one which is acting as a raw material. The new product will exhibit the same properties and qualities as the previous one, i.e., materials will remain in a continuous cycle of life. In general, a huge amount of this waste is composed of plastics whose versatility and wide range of properties makes it difficult to get a competitive alternative in terms of costs. Some biopolymers being investigated by scientists and industry are biodegradable, and specifically, obtained from agricultural and food processing waste. Chitosan is one of the most studied biopolymers due to its biocompatibility, biodegradability, adhesivity, and bioactivity. Chitosan is the second most abundant biopolymer in the world after cellulose; this arouses researchers’ interest in fabricated novel and sustainable materials based on it. On the other hand, its low cost also makes it a good choice of material [1]. The chitosan is used in a wide range of applications and industries, related to agriculture, pharmacy, medicine, food, or textile among others [2,3,4,5,6]. Nonetheless, new developments involve biomedicine, biotechnology, wastewater treatment, catalysis, packaging, or bioimaging which are essential for a new sustainable era where chitosan can provide versatility, recyclability, and low cost. The nature and properties of chitosan lend themselves to sustainability criteria, due to its biodegradability, bioactivity, or the obtaining method, but there are also some specific applications related to sustainability where the chitosan can play an important role, in terms of efficiency, yield, and cost. Probably, the most important applications of chitosan in this field are associated with wastewater treatment, absorption of pollutants, or their uses as a chelation agent, an antiviral agent, or a substitute material in the paper industry [7]. Some of these recent advances involve chitosan for the preparation of composites or functionalized materials, such as aerogels based on chitosan and soot.

Chitosan biopolymer can be functionalized by several function groups. Functionalization can be grafting, addition, coupling, crosslinking, etc. [8]. These were tested for the adsorption of dyes and other pollutants, such as naphthalene, showing interesting results [9]. The combination of chitosan with other materials such as collagen can also increase the range of its features [10]; for instance, the preparation of tailored scaffolds which allows adapting their properties to clinical demand [10]. 

The preparation of nanoparticles or nanocomposites also contributes to the circular economy, as a lower amount of raw materials is necessary for developing a specific application-based sustainable materials. Nanocomposites with magnesium show great activity against different pathogens developed in many plants, such as *Acidovorax oryzae* and *Rhizoctonia solani* which both are rice pathogens [11]. A greater surface area can be obtained through the production of thin films reducing the amount of raw materials and consequently the volume of waste after use, but keeping the same properties of the original films. Some of these developments can be carried out using chitosan, specifically for the food packaging [12]. This mini review collects some of the most relevant points that chitosan can offer for sustainable development. The new trends in science are focused on green chemistry and the circular economy; this manuscript collects brief goals, methods, and applications which are essential for understanding the importance of chitosan for new generations.

### 1.1. Chitosan as a Renewable Material

#### 1.1.1. Chitosan as a Biomaterial

Chitosan is obtained through the deacetylation of chitin, which is one of the most abundant biomaterials after cellulose. This one is a polysaccharide which can be found in crustaceans, insects, or fungi (Table 1) [13]. Chitin is considered a linear long-chain homopolymer which is composed of N-acetyl glucosamine, and can develop three polymorphic forms known as α-, β-, and γ-chitin [14].

**Table 1:** Some of the main chitin sources and percentages [13].

Source	Percentage (%)
Shrimps	30–40%
Squids	20–40%
Krill	20–30%
Crabs	15–30%
Fungi	10–25%
Insects	5–25%
Oysters	3–6%
Clams	3–6%

**Figure 1:**
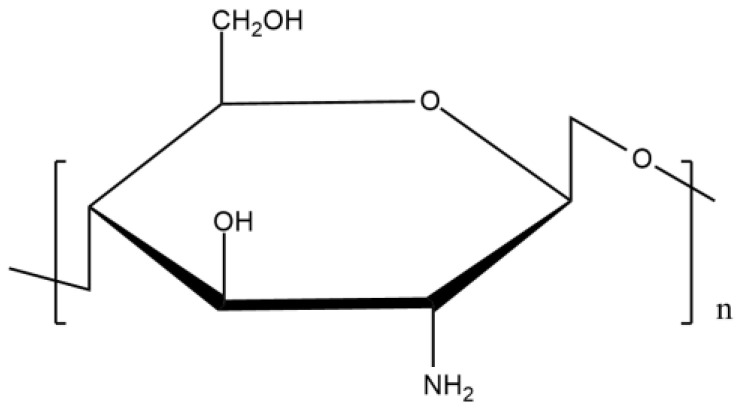
Chemical structure of chitosan.

Commercial chitosan (Figure 1) is composed of D-glucosamine and N-acetyl glucosamine and is produced by the partial deacetylation of chitin. This reaction carries out the change of acetamido groups into amino groups. There are three kinds of this biopolymer depending on its molecular weight: low molecular weight, high molecular weight, and oligochitosans [15].

#### 1.1.2. General Features and Properties of Chitosan

The main properties which can contribute to a sustainable development that are exhibited by the chitosan are non-toxicity, biodegradability, and biocompatibility. Nevertheless, there are other interesting properties and characteristics which explain its versatility which can be deduced from Table 2.

**Table 2:** General properties of chitosan [16,17].

Property	Conditions	Use	References
Solubility	Dilute acids (pH < 6). Insoluble in organic solvents and water	Water treatment	[18,19]
Activity		Antibacterial, antifungal mucoadhesive analgesic, and hemostatic properties	[20,21,22]
Degradation	Depends on molecular weight and deacetylation degree		[18,23]
Biocompatibility	Physiological medium	Biomedical applications	[7,24]
Chelating properties	Capability to bind and adsorb diverse ions	The removal of heavy metals and dyes from wastewater	[25,26]
Biodegradability	Biodegradable to normal body constituents		[24,27,28]
Hemostatic		Stop a hemorrhage	[29,30]
Catalyst	Accelerates the formation of osteoblast		[31]
Fungicide		Stopping the development of fungi	[32,33]
Spermicidal		Reduce the mobility of spermatozoa	[34]
Anticholesteremic		Reducing agent cholesterol	[35,36]
Anticancer		Inhibiting the development of cancer cells	[37]
Conductivity	Ionic conductivity		[38,39]
Flocculating agent	Interactions with negatively charged molecules	Water treatment	[40]
Thickener		Increase the viscosity	[41]
Polyelectrolytes	Acidic medium		[42]
Adsorption		Separation and filtration	[43,44,45]
Clarifying agent		Immobilization of enzymes	[46]

From the presentation of Table 2, it can be deduced that chitosan is a sustainable material as it is biodegradable and non-toxicity [47]. Another important reason for using chitosan is the presence of a large number of hydroxyl and amino groups in its structure which are suitable for chemical modifications [48]. This fact and the wide versatility of chitosan makes this material especially interesting for the preparation of suspensions, composites, functionalized materials, or (nano)hybrids for diverse eco-friendly purposes and applications. The interesting polymorphic behavior exhibited by the chitosan [49], together with the molar mass and degree of deacetylation, mainly defines its mechanical properties. The molar mass will also play an important role for other properties such as degradation degree or antibacterial activity as these are strongly affected by the changes in molar mass.

On the other hand, the degree of deacetylation is associated with the content of acetamide groups of polymeric chains. These groups will strongly affect the final features and properties of the chitosan, in particular its capacity to be biodegradable and its immunological activity. The deacetylation degree is defined between 50 and 99%, its content depends on the preparation methods. The deacetylation degree must be higher than 50% for the chitosan; below that value, it is considered chitin [18]. Some of the most important uses of chitosan are associated with biomedical applications. Nevertheless, new developments related to chitosan focus on agriculture, food packaging, textiles, or environmental applications [50]. The solubility of the chitosan depends on the medium being used to dissolve it; in acid mixtures with water, it is soluble, but it is insoluble in common organic solvents [51,52]. The reason for its solubility can be explained due to the presence of amino groups that transforms chitosan into a base, whose protonation produces a polyelectrolyte [53]. The presence of different functional groups is responsible for the reactivity and the flexibility of this polycationic polymer [54]. Chitosan biofilms show a semi-crystalline behavior, together with high hydrophobicity and little flexibility [55].

#### 1.1.3. Chitosan as an Ecofriendly Biopolymer and Its Applications

Chitosan is considered a natural biopolymer; it has received remarkable attention from the scientific community due to the fact that it can be easily biodegraded. Its residues are not toxic and can be easily eliminated and biodegraded by nature [7]. One of the most important problems associated with the raw materials is that these are limited, but chitosan is the most abundant biopolymer after cellulose. Furthermore, chitosan exhibits a great biocompatibility, limited by its low solubility which can be solved through chemical modifications and hydrolysis. Chitosan is a bioactive material which can be modulated and used in many applications [56]. Some of these applications are associated with biomedical purposes such as drug delivery systems, scaffolds, or membranes. Nevertheless, there are other important uses such as in the textile industry, wastewater treatments, agriculture, food, packaging, personal care, and biotechnology, among others. The adsorbent properties of chitosan are very useful for removing different heavy metal ions accumulated in water and derived from industrial processes such as Pb^2+^, Hg^2+^, and Cu^2+^, among others [57]. These can be accumulated inside the body and produce numerous diseases [58]. Chitosan can contribute to the agriculture by improving the harvest and productivity, being an ecofriendly material. It is used as a coating for seeds, enhancing the properties of the plants and the obtained products in terms of shelf life. This use as fertilizer is especially useful for plant protection as it can stimulate the plant defense, but it can also act as an antibacterial and antimicrobial agent [59]. Thus, chitosan acts as a plant growth-promoting agent and plant protector [60]. For that reason, it is considered a pesticide by several countries. The antioxidant properties of chitosan, together with its antimicrobial features, are suitable for the production of films for food packaging. The preparation of hybrid materials with chitosan allows modifying the permeability of those films depending on the requirements [2]. The chitosan can also be used as a food additive, dietary fiber, and functional ingredient [61,62].

## 2. Sustainable Production

### 2.1. Chitin Extraction

**Figure 2:**
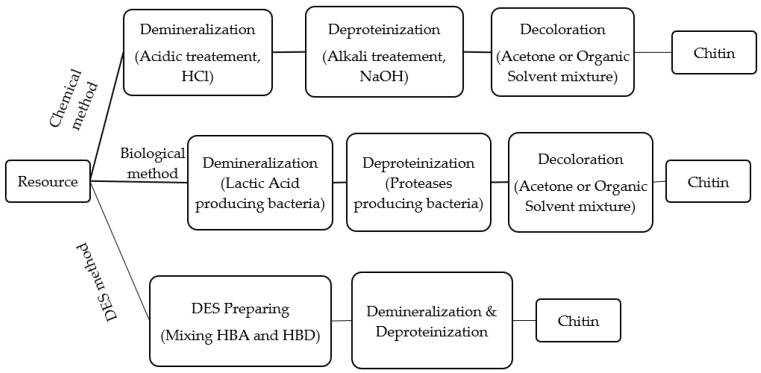
Extraction of chitin. DES: deep eutectic solvents; HBA: hydrogen bond acceptor; HBD: hydrogen bond donor.

The extraction of chitin is necessary for the production of chitosan such as it was previously explained. A huge amount of chitin is obtained from crustaceans, but there are multiple advances in its production through insects or fungi and bacteria, thus avoiding the use of animal derivatives [63]. In general, the extraction requires several steps starting with the removal of mineral salts and proteins (Figure 2). It is commonly carried out chemically, using acids and bases, which is not a sustainable process. These processes can destroy some properties of chitosan, reducing its versatility. Currently, there are multiple advances in natural deep eutectic solvents which could replace the hazardous solvents and preserve the features of chitin. There is another option based on the use of microorganisms for the extraction of chitin known as a biological method [64]. In general, these methods are especially indicated for the treatment of fungi and bacteria whilst chemical processes are related to the treatment of crustaceans. After removing the minerals and proteins, chitin requires a depigmentation process which is generally performed using oxidizing agents. The use of the enzymes could be a feasible way for removing the proteins, which can reduce the degree of depolymerization in comparison with traditional methods. That chitin also showed a better solubility in water probably due to a lower crystallinity of the product [65]. The specific use of the trypsin also induces the depigmentation, reducing the steps involved in the extraction of chitin [66]. There is a lot of ground to cover in terms of sustainability around processes for the extraction of chitin associated with environmental pollution, loss of chitin properties, and costs. One of the main consequences of this extraction is the polluted wastewater, which needs to be treated.

### 2.2. Chitosan Production

**Scheme 1:**
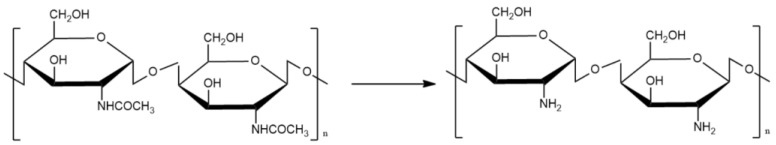
Deacetylation of chitin.

The production of chitosan requires the deacetylation of chitin; this process can be modulated through concentration, temperature, and time [7]. Scheme 1 shows the changes produced in chitin after being transformed into chitosan.

The traditional method to obtain chitosan from chitin was reported in 1980, which promotes a high deacetylation due to rapid reaction rates at reduced temperatures [67]. There are different ways to carry out the deacetylation such as alkali treatment, the use of enzymes, or a steam explosion [16,68,69]. The degree of deacetylation will define the spectra of properties of the chitosan in terms of features such as solubility, viscosity, or biodegradability, etc. [70]. There are numerous alternatives where the energy consumption can be reduced, contributing to a green chemistry. Those methods explore the use of microwaves and ultrasonic waves in the deacetylation process. The use of ultrasonic waves leads to enhancing the reactivity of the deacetylation process [71]. Some of the new approaches are displayed in Table 3, showing some of the most interesting advances related to the sustainable production of chitosan.

**Table 3:** New methods for the production of chitosan.

Treatment	Disadvantages	Advantages	Reference
Trypsin (crustaceans)	Only for deproteination step	Depigmentation of treated material	[66]
Streptomyces griseus (crustaceans)	Only for deproteinization	Better solubility	[65]
Bacillus mojavensis A21 or Balistes capriscus (crustaceans)	Deproteinization requires NaOH	Optimized process	[72]
Rhizopus oryzae (fungi)	Fermentation	Cheap, low energy consumption, and soft conditions	[73]

### 2.3. Circularity in the Chitosan Production

The traditional methods can also be adapted, at least partially, trying to get a sustainable production of chitosan. For that purpose, it is necessary to reduce the energy consumption by reusing the hazardous reagents. The recovery of sodium hydroxide used in the extraction of chitosan was reported in studies. The sodium hydroxide is part of wastewater and could be treated using ultrafiltration and nanofiltration membranes recovering the sodium hydroxide for a new cycle of life [74,75]. The reuse of sodium hydroxide can contribute to a decrease the environmental pollution and reducing the cost of the process, i.e., a lower amount of sodium hydroxide will be required. There were also reports for the preparation of chitosan at ambient temperature, following the general procedure of demineralization, deproteinization, and decolorization [76]. This fact could also be quite interesting, due to the reduced energy consumption. Thus, involving circularity in the production of chitosan can be very beneficial and economically better.

## 3. Applications of Chitosan for Sustainable Development

**Figure 3:**
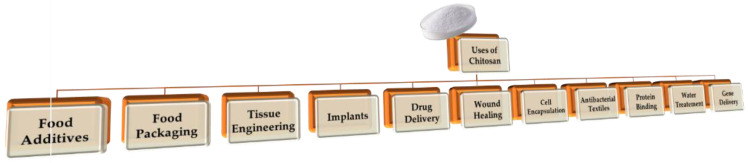
Different uses of chitosan.

Chitosan can contribute to sustainable development through its applications and uses. This review tries to expose some of the most important applications related to the contribution of chitosan to a circular economy and sustainability. Figure 3 depicts the diversified application of chitosan.

### 3.1. Sustainable Use of Chitosan for Food Packaging and in Agriculture

Many biopolymers are being implemented in different coating materials due to their excellent properties in terms of degradability and compatibility; these biopolymers include gums, starch, proteins cellulose, lipids, and their derivatives [77,78,79,80,81,82,83]. In this sense, chitosan is a promising material for that purpose due to several reasons associated with its biocompatibility and abundance [84,85]. The use of the chitosan in films can also provide other superiorities because of its antibacterial and antioxidant properties [86,87,88,89]. In general, chitosan is used in combination with other polymers due to some of its drawbacks associated with its low mechanical properties. Another important problem associated with chitosan is related to its water sensitivity [90]. The preparation of blends can diminish these problems, thus obtaining films with a wide range of properties. The miscibility problems between the mixtures of polymers can reduce the spectra of possibilities, but in general, the preparation of these films is easy and cheap. The preparation of these systems could be a good alternative regarding traditional films based on oil derivatives [91]. Table 3 displays some of the most promising blends of chitosan, based on the mixtures with other biopolymers. There are other mixtures with synthetic polymer of chitosan that are not included in this review, as those do not fit the sustainability criteria of the present review. Numerous composites of chitosan have been fabricated with graphene, carbon nanotubes, activated carbon, and metal nanoparticles [92,93,94,95]. One study suggests that poly(L-lactic acid)-ZnO multilayered with cationic chitosan and anionic β-cyclodextrin can be used as a promising material in applications for the active packaging of food [96]. A novel bilayer food packing film of Ag-Metal−organic framework loaded p-coumaric acid modified chitosan (P-CS/Ag@MOF) or chitosan nanoparticles (P-CSNPs/Ag@MOF) and polyvinyl alcohol/starch (PVA/ST) was fabricated. The bilayer composite film revealed a relatively smooth surface and higher tensile strength (27.67 MPa). The P-CS/Ag@MOF bilayer films displayed better oil resistance and oxidation resistance, and the bilayer film had good UV-blocking properties and transparency [97]. The diverse blend composites of chitosan have been developed with various natural antimicrobial compounds and have been applied for antimicrobial food packaging; such antimicrobial compounds include thyme oil, spirulina, oregano essential oil, nisin, apple peel polyphenols, bamboo vinegar, cinnamon essential oil, custard apple leaves, plum peel extract, etc. [98,99,100,101,102,103,104]. The antibacterial nanofiber films were fabricated using gelatin, chitosan, and 3-phenyllactic acid (PLA) by electrospinning. Under acidic conditions, chitosan and PLA interacted and formed hydrogen bonds, which decreased the crystallinity of the nanofiber films. The nanofiber film had the best thermal stability, water stability, water vapor permeability, and more effective antibacterial effects against *Salmonella enterica Enteritidis* and *Staphylococcus aureus*, suggesting that the nanofiber film mat can be used as an active food packaging [105]. Similarly, Wang et al. discussed various chitosan and gelatin edible films, their synthesis strategies including casting, electrospinning, and thermoplastic method, and their properties in their review, thus highlighting importance of chitosan-based food packing films [106]. In Argentina, chitosan is produced from the waste of the shrimp industry; the synthesized chitosan has similar physicochemical properties to those of analytical grade chitosan. The chitosan coatings applied to processed lettuce at harvest increased nutritional quality and reduced microbiological contaminants in minimal processed lettuce [107]. Panda et al. fabricated ferulic acid-modified water-soluble chitosan and poly(γ-glutamic acid) polyelectrolyte multilayers films. These film surfaces possessed a reduced amount of protein adsorption; thus, these can be used as a potential good biomaterial for biomedical purposes to intensify the bio-active surface [108], thus prompting the concept of circularity and sustainability. Table 4 and Table 5 show the effects of some films over the food due to the use of chitosan which could modify its properties.

**Table 4:** Selection of blends of chitosan with other biopolymers for food packaging.

Biopolymer	Chitosan	Characteristics	Reference
Pectin (2% *w*/*v*)	2% *w*/*v*	Good mechanical properties. Antimicrobial activity.	[109,110]
Carboxymethyl cellulose (1–2% *w*/*v*)	1% *w*/*v*	Better mechanical properties and permeability. Antioxidant and antimicrobial activity.	[111,112,113]
Gum arabic (1.5% *w*/*v*)	1.5% *w*/*v*	High elasticity. Antioxidant and antimicrobial activity.	[114,115]
Cassava starch (3% *w*/*v*)	0.5% *w*/*v*	Antibacterial activity.	[116]
Corn starch (5% *w*/*v*)	(1, 2, 3, and 4% *w*/*v*)	Higher tensile strength and elasticity. Lower permeability.	[117]
Rice starch (2% *w*/*v*)		Better barrier properties.	[118]

**Table 5:** Effects of films based on chitosan over food.

Blend	Food	Effects	References
Chitosan-glycerol film (Good mechanical and barrier properties. Stability)	Strawberry	Better preservation effect than the commercially available PE films.	[119]
Gelatin/chitosan film with nanocarriers (Fe^III^-HMOF-5) (Good results in mechanical properties and permeability)	Apple cubes	High content of nanocarriers allows the preservation of apple cubes during 5 days.	[120]
Chitosan films (modified with mango leaf extract) (Higher hydrophobicity and tensile strength)	Cashew nuts	High oxidation resistance.	[121]
Chitosan/gelatin film with silver nanoparticles (Better hydrophobicity and antibacterial properties)	Red grapes	Antimicrobial properties and high oxidation resistance.	[122]
Polyurethane/chitosan/nano ZnO composite film (Better mechanical properties, low permeability)	Carrot	Better shelf life than polyethylene film	[19]
Pullulan/chitosan film (good barrier to O_2_)	Papayas	Maintained the physiological and nutritional attributes. High shelf life.	[123]
Chitosan-TiO_2_ nanocomposite film (Better tensile strength and barrier properties)	Tomatoes	Delay the ripening process and extend the storage life.	[124]
Cellulose/chitosan/polypyrrole film	Cherry tomatoes	Possess good antioxidant, antibacterial, and barrier properties	[125]
Baicalin-liposomes loaded polyvinyl alcohol-chitosan electrospinning nanofibrous films	Mushrooms	Possessed effective antibacterial properties, non-cytotoxicity, and preservation performance	[126]
Active packaging films based on chitosan and sardinella protein isolate	Shrimps	Good antioxidant and antibacterial activities	[127]
ε-polylysine/chitosan nanofibers	Chicken	Inhibiting *Salmonella typhimurium* and *Salmonella* *enteritidis* on chicken	[128]
Chitosan films embedded with Apricot (*Prunus armeniaca*) oil	Bread	Better antioxidant, mechanical, and antimicrobial properties	[129]
Zein active film containing chitosan nanoparticle encapsulated with pomegranate peel extract	Pork	Addition of chitosan nanoparticle can increase the thermal stability of zein active film Film can inhibit the growth of *Listeria monocytogenes* on pork	[130]
Mahua oil-based polyurethane/chitosan/nano ZnO composite films	Carrot	Excellent anti-bacterial properties against Gram positive and Gram-negative bacteria Increase shelf life of carrot	[131]
Carboxymethyl chitosan (CMCh)-peptide conjugates	Blueberry	Extend the shelf-life of blueberry	[132]
Chitosan-based biodegradable bags	Palmer’s mango	Effective in delaying ripening and preserving the quality	[133]
Composite films based on chitosan and syringic acid	Quail eggs	Films exhibited higher density, water solubility, good preservation effect	[134]
Films based on quaternary ammonium chitosan, polyvinyl alcohol, and betalains-rich cactus pears (*Opuntia ficus-indica*) extract	Shrimp	Enhanced the UV–vis light barrier, elongation-at-break, and antioxidant, antimicrobial and ammonia-sensitive properties	[135]
Chitosan coating with vacuum packaging	Beef	Extend the shelf life of beef Inhibited *S. aureus*	[136]
Chitosan coatings	Lettuce	Improve quality and extend shelf-life of minimally processed lettuce	[107]
Chitosan films incorporating litchi peel extract and titanium dioxide nanoparticles	Watercored apple	Coating treatment significantly inhibited respiration rate, weight loss, and softening	[137]
Polylactic acid/chitosan films	Indian white prawn	Antimicrobial properties	[138]
Chitosan-Gelatin (CHI-Gel) based edible coating incorporated with longkong pericarp extract (LPE)	Shrimp	Edible coating as a natural antioxidant, antimicrobial activity and inhibiting melanosis, retain the quality and extend the shelf-life	[139]
Pink pepper residue extracts incorporated in a chitosan film	Salmon fillets	Shelf-life of the skinless salmon fillet could be extended by 28 days	[140]
Chitosan film incorporated with citric acid and glycerol	Green chilies	Improved mechanical, thermal, and antioxidant properties of the film were and increased shelf life	[141]

The chitosan can act as protector, coating material, stimulator of the growth, nutrient, fertilizer, or pesticide in agriculture. It was also observed that the use of chitosan can increase productivity. Furthermore, the use of chitosan could replace some dangerous chemicals used as compounds of fertilizers in agriculture, protecting soil, aquifers, and ecosystems [142]. It was reported that excellent antimicrobial activity was observed in chitosan against many viruses, bacteria, and fungi. Nevertheless, its activity is higher against fungi than bacteria. In general, the chitosan seems to inactivate the replication of viruses [143]. Moreover, it is considered a potent elicitor which can induce plant defense against diseases [144]. Table 6 shows some of the effects observed of chitosan over some fruits and vegetables.

**Table 6:** Effects of chitosan and derivatives over some products.

Material/Use	Plant	Effects	Reference
Chitosan with copper	Tomato	Plant defense (Enzymatic and anatomical changes).	[145]
Seed-priming with chitosan	Cucumber	Disease protection and enhanced plant growth.	[146]
Foliar application of chitosan	Sweet pepper	Enhancement of the adverse effects of salinity and improved the growth and yield.	[147]
Chitosan solution (using a hand sprayer)	*Dracocephalum kotschyi*	Increase of antioxidant enzyme.	[148]
Chitosan (foliar spray or pre-sowing seed treatments in Cd-stressed plants)	Pea	Improvement in growth, photosynthetic pigments, and reduction in oxidative damage.	[149]
Chitosan (protective spray)	Mango (Amrapali and Dashehari)	Reduced malformation of mango.	[150]
Chitosan nanoparticles	Durum wheat	Increase the leaf antioxidant pool.	[151]
Chitosan oligosaccharide (COS)	Tea plant (*Camellia sinensis*)	Improved the antioxidant enzyme activities and the content of chlorophyll and soluble sugar.	[152]
Chitosan nanoemulsion containing allspice essential oil	Maize	Preserved maize samples from aflatoxin B1 and lipid peroxidation.	[153]
Chitosan nanoparticles loaded with garlic essential oil	Wheat, oat, and barley	As a seed dressing agent found to have antifungal activity against *Aspergillus versicolor*, *A. niger*, and *Fusarium oxysporum.*	[154]
1.5% chitosan solution treatment	Berry	Inhibit postharvest berry abscission of the ‘Kyoho’ table grapes.	[155]
Preharvest chitosan sprays	Muskmelons	Induced suberin polyphenolic deposition at wound sites during healing thus promoted wound healing and reduced disease development.	[156]
Chitosan film containing *Akebia trifoliata* (Thunb.) Koidz. peel extract/montmorillonite	*A. trifoliata* fruits	Significant effect on the delaying crack and mature of the fruits.	[157]
Chitosan-based nanoencapsulated *Foeniculum vulgare* Mill. essential oil	*Sorghum bicolor*	Significantly preserved the nutritional and sensory characteristics of *S. bicolor* seeds.	[158]
Encapsulated peppermint essential oil in chitosan nanoparticles	*-*	Biological efficacy against stored-grain pest control.	[159]

### 3.2. Sustainable Applications of Chitosan in Purification of Water, Paper-Making, and Green Chemistry

The chitosan is a good flocculant for water treatment, especially indicated for organic matter, suspended solids, and ions (metals). Furthermore, the deposition rate is stimulated when chitosan is used [160]. It is used over oil spills as it can preserve the integrity of the oil mass. Its properties are also indicated for anionic waste where the chitosan can remove the metal ions of the acid solutions. Some of the most attractive features of chitosan regarding other flocculants are associated with its biodegradability and its adsorption and flocculating ability, which show excellent results with oils [7]. However, there are many other pollutants where the chitosan shows interesting results as can be observed in Table 7. Chitosan and its composites demonstrate excellent adsorption properties for diversified environmental contaminates ranging from organic pollutants to metal ions [47,161,162,163,164,165]. The mechanism for the adsorption of toxic pollutants by chitosan and its composites involves various types of interactions such as electrostatic, hydrogen bonding, π-π bonding, etc. The chitosan and its composites had several hydroxyls and amino and carboxylic groups which are very helpful for such interactions, thus making it more adsorbent.

**Table 7:** Examples of pollutants removed by chitosan and derivatives.

Pollutant	Adsorbent	Efficiency	References
Tetracycline	Chitosan/poly (vinyl alcohol) nanofibers	102 mg/g (maximum adsorption capacity)	[166]
Ciprofloxacin	Chitosan/biochar hydrogel	36.72 mg/g (uptake capacity)	[167]
Tetracycline	Magnetic polymer nanocomposite was fabricated using chitosan, diphenyl urea, and formaldehyde	168.24 mg/g (maximum adsorption capacity)	[168]
Tetracycline	Nanocomposite of chitosan/thiobarbituric acid/malondialdehyde-Fe_3_O_4_	215.31 mg/g (highest adsorption capacity)	[169]
Antibiotics	Chitosan-grafted SiO_2_/Fe_3_O_4_ nanoparticles	100.74 mg/g (theoretical adsorption capacity)	[170]
Ketoprofen	Chitosan/Zr-MOF (UiO-66) composite	Maximum adsorption capacity of 209.7 mg/g	[171]
Tetracycline	Nitrilotriacetic acid modified magnetic chitosan-based microspheres	Adsorption capacity of 373.5 mg g^−1^	[172]
Congo red	Chitosan nanoparticles	99.96%	[173]
Methylene blue	Chitosan/κ-carrageenan/acid-activated bentonite composite membranes	Maximum adsorption capacity for methylene blue was 18.80 mg/g	[174]
Azo dyes	Glass beads coated with chitosan	Maximum adsorption capacity of the column packed with GBCC was 108.7 mg g^−1^.	[175]
Methyl orange	Chitosan-lysozyme biocomposite	Maximum adsorption capacity for MO was 435 mg/g	[176]
Methylene blue	Bivinylbenzene cross-linked chitosan/maleic anhydride polymer	Adsorption capacity for MB 503 mg/g	[177]
Acid orange 7 (AO7, monovalent), Acid red 13 (AR13, divalent), and Acid red 27 (AR27, trivalent) dyes	Chitosan–magnetite gel microparticles	Acid Orange 7 (AO7, monovalent), Acid Red 13 (AR13, divalent), and Acid Red 27 (AR27, trivalent) dyes with maximum adsorption capacities, *Q*_max_, of 1.71, 1.55, and 1.13 g-dye/g-dry adsorbent, respectively	[178]
Methyl orange dye	Fe-loaded chitosan film	Maximum adsorption capacity 205 mg g^−1^	[179]
Methyl orange dye	Chitosan/carbon/Fe_3_O_4_	Maximum adsorption capacity was 425 mg g^−1^	[180]
Disperse blue 367	Magnetic/chitosan/graphene oxide	Adsorption capacity of 298.27 mg/g	[181]
Reactive orange 16 dye	Chitosan tripolyphosphate/TiO_2_ nanocomposite	Adsorption capacity was 618.7 mg/g	[182]
Acid red 88	Phosphorylated chitosan	Adsorption capacity was 230 mg g^−1^	[183]
Methylene blue	Poly(glycerol sebacate)/chitosan/graphene oxide nanocomposites	Adsorption capacity was 129 mg/g	[184]
Methylene blue	Magnetic sodium ferrosilicate/carboxymethyl chitosan composite	Adsorption capacity was 515.0 mg/g	[185]
Malachite green (MG), reactive red (RR), and direct yellow (DY) dyes	Chitosan	Adsorption capacities 166 mg/g for dye MG, 1250 mg/g for dye RR and 250 mg/g for dye DY	[186]
Methyl orange	Chitosan crosslinked with metal-organic framework (MOF-199)@aminated graphene oxide aerogel	Maximum adsorption capacity for methyl orange 412 mg/g	[187]
Reactive orange 16	Chitosan-polyvinyl alcohol/fly ash (m-Cs-PVA/FA)	Adsorption capacity of m-Cs-PVA/FA for RO16 dye removal was 123.8 mg/g	[188]
Methyl orange and methylene blue	Graphene oxide-chitosan composite	Maximum adsorption amounts of MO and MB were 543.4 and 110.9 mg/g	[189]
Phenol, BPA, and 2,4-DCP	Chitosan modified nitrogen-doped porous carbon composite	Maximum adsorption capacity for phenol, BPA, and 2,4-DCP was 254.45, 675.68, and 892.86 mg g^−1^	[190]
Sunset yellow	Chitosan	Maximum adsorption capacity 1432.98 mg g^−1^	[191]
Allura red	Luffa-chitosan crosslinked with glutaraldehyde (LCsG) and epichlorohydrin (LCsE)	LCsG and LCsE presented maximum capacities of 89.05 mg/g and 60.91 mg/g.	[192]
Brilliant blue	Chitosan	Maximum adsorption capacity 814.27 mg/g	[191]
Tartrazine	Chitosan	Maximum adsorption capacity 1065.55 mg/g	[191]
Acid blue-25	Chitosan/porous carbon composite modified in 1-allyl-3-methyl imidazolium bromide ionic liquid	Maximum adsorption capacity 3333.33 mg/g	[193]
Morphine, codeine, ephedrine, amphetamine, and benzoylecgonine	Magnetic chitosan-graphene oxide-ionic liquid ternary nanohybrid	Adsorption capacity for morphine, codeine, ephedrine, amphetamine, and benzoylecgonine (7.2, 8.4, 9.2, 5.8, and 11.2 mg g–1, respectively)	[194]
Tartrazine	Chitosan/polyaniline composite	Maximum adsorption capacity of 584.0 mg/g	[195]
Acetaminophen	Polyaniline with chitosan	Adsorption rate of 385.25 mg.g^−1^	[196]
Anthocyanins	Chitosan beads	Adsorption capacity was 216 mg g^−1^	[197]
Tetracycline	Zirconium-loaded chitosan modified by perlite (Zr/Cht/Pt) composites	Maximum adsorption capacity of 104.17 mg/g	[198]
Levofloxacin, tetracycline hydrochloride, and sulfamethoxazole	Chitosan	Adsorption capacity of levofloxacin, tetracycline hydrochloride, and sulfamethoxazole were 26, 22, and 67 mg/g	[199]
17α-ethinylestradiol	Graphene oxide, magnetic chitosan, and organophilic clay composite	Maximum adsorption capacity was 50.5 mg/g	[200]
Tartrazine	Surfactant-ionic liquid bi-functionalization of chitosan beads	Adsorption capacity was found to be 45.95 mg/g	[201]

The chitosan also showed good results associated with ions, as it can be observed in Table 8. These are only some examples of the good results that can be achieved.

**Table 8:** Examples of chitosan for removing ions.

Ion	Adsorbent	Efficiency	References
Cr (VI), Cu (II), and Co (II)	Polyethylenimine-grafted chitosan electrospun membrane	138.96, 69.27, and 68.31 mg/g for Cr(VI), Cu(II), and Co(II), respectively (maximum adsorption capacities)	[202]
Cu^2+^ and Cr^6+^	Zeolitic imidazolate framework-67 modified bacterial cellulose/chitosan composite aerogel	200.6 mg/g and 152.1 mg/g, for Cu^2+^ and Cr^6+^, respectively (adsorption capacities)	[203]
Cu^2+^	Monodispersed chitosan microspheres	75.52 mg/g (adsorption capacity)	[204]
Pb^2+^, Cu^2+^, and Cd^2+^	Physically crosslinked chitosan/sodium alginate/calcium ion double-network hydrogel	176.50 mg/g, 70.83 mg/g, and 81.25 mg/g for Pb^2+^, Cu^2+^, and Cd^2+^, respectively (adsorption capacities)	[205]
Cu^2+^, Pb^2+^, and Cd^2+^	Chitosan-coated argillaceous limestone	64.11 mg/g, 217.4 mg/g, and 52.48 mg/g for Cu^2+^, Pb^2+^ and Cd^2^, respectively (maximum adsorption capacities)	[206]
Cr(VI)	Terylene carbon-dots modified chitosan non-woven fabrics	Maximum adsorption capacity was 203 mg/g	[207]
Pb^2+^	Zeolitic imidazolate framework-8 (ZIF-8) on carboxymethyl chitosan beads	Maximum adsorption capacity of 566.09 mg/g	[208]
Cd^2+^	Cellulose/chitosan composite spheres loaded with nZVI	Maximum adsorption up to 110.3 mg/g	[209]
Cu^2+^ and Ni^2+^	Tripolyphosphate-crosslinked-chitosan-modified montmorillonite	Adsorption capacity for Cu^2+^ and Ni^2+^ 0.56 and 0.44 mmol/g	[210]
Cr^4+^	Chitosan-lysozyme biocomposite	Maximum adsorption 216 mg g^−^^1^	[176]
Pb^2+^ and Cd^2+^	Chitosan/Mg-Al-layered double hydroxide nanocomposite	Maximum capacities were 333.3 mg/g for Pb^2+^ and 140.8 mg/g for Cd2^+^, respectively.	[211]
Arsenic	Silica-stabilized magnetic chitosan Beads	Maximum adsorption capacity 1.699 mg/g	[212]
Cr(III) and Cr(VI)	Iron oxide/carbon nanotubes/chitosan magnetic composite film	Maximum adsorption capacity for Cr(III) of 66.25 mg/g and for Cr(VI) of 449.30 mg/g	[213]
Cu(II)	Chitosan-coated magnetic nanoparticles	Maximum adsorption capacity was found to be 236.7 mg/g	[214]
Cr(VI)	Nano-graphene oxide-assisted hydrotalcite/chitosan biocomposite	Maximum adsorption capacity of 42.64 mg/g	[215]
Pb^2+^ and Hg^2+^	Schiff base based on porous chitosan-glutaraldehyde/montmorrilonite nanoparticles modified with 3-aminopropyl triethoxysilane	Maximum adsorption capacity of Pb^2+^ and Hg^2+^ were 32.786 and 30.395 mg/g	[216]
Re(VII)	Chitosan-silica composite containing Mo-imprinted cavities	Adsorption capacity of 368.8 mg g^−1^	[217]
Uranium	Chitosan-grafted adenosine 5′-monophosphate foam	Adsorption capacity of 311 mg/g	[218]
Li^+^	H_4_Mn_5_O_12_/chitosan	Adsorption capacity reached 11.4 mg/g	[219]
Fluoride	Zirconium (IV)-impregnated magnetic chitosan graphene oxide	Adsorption capacity was 8.84 mg/g	[220]
U(VI)	Chitosan-based aerogel	U(VI) adsorption capacity of 160 mg/g	[221]
Au(III)	Chitosan functionalized with N,N-(2-aminoethyl)pyridinedicarboxamide	Maximum adsorption capacity of 659.02 mg/g	[222]
Cr(IV)	Chitosan composite	Adsorption capacity was 18 mg/g	[223]
Cu(II)	Benzothiazole functionalized chitosan	Maximum copper adsorption capacity of 1439.7 mg/g	[224]
Cr(IV)	Chitosan-crosslinked-poly(alginic acid)	Maximum adsorption capacity 26.49 mg/g	[225]
Pb(II)	Ninhydrin-functionalized chitosan	Maximum adsorption capacity of 196 mg/g Pb(II) ions	[226]
Co^2+^ and Sr^2+^	Fibrous chitosan biosorbent	Adsorption capacity of fibrous chitosan for Co^2+^ and Sr^2+^ was 31.3 mg g^−1^ and 20.0 mg g^−1^	[227]
Au(III)	Benzothiazole-modified chitosan	Maximum adsorption capacity of 1072.22 mg/g	[228]
Cu(II)	Polyacrylamide-modified kaolin enhances adsorption of sodium alginate/carboxymethyl chitosan hydrogel beads	Adsorption capacity of the adsorbent was 5.5157 mg/g	[229]
Ag(I)	Chitosan-coated magnetic silica core-shell nanoparticles	126.74 mg/g	[230]
Cu^2+,^ Fe^3+^ and Pb^2+^	Chitosan	Maximum adsorption capacity Cu^2+,^ Fe^3+^, and Pb^2+^ were 462 270 mg/g, 934 mg/g	[199]
Sr^2+^	Carboxymethyl chitosan gel	Maximum adsorption capacity can reach 144.73 mg/g	[231]
As(III)	MnO_2_-strengthened WTRs-chitosan beads	Adsorption capacity of 36.911 mg/g	[232]
As(III), Cd(II), Cu(II), and Pb(II)	Chitosan bead-supported MnFe_2_O_4_ nanoparticles	As(III), Cd(II), Cu(II), and Pb(II) was achieved maximum adsorption capacities of 9.90, 9.73, 43.94, and 11.98 mg/g	[233]

Chitosan can be used for paper manufacture due to its mechanical properties which can provide better resistance to recycled paper, reducing the consumption of chemical additives [234]. Table 9 displays the various roles of chitosan in paper production.

**Table 9:** Effects of chitosan in paper production.

Material/Use	Paper Application	Effects	Reference
Nanoparticles with chitosan and starch	Old corrugated containerboard (OCC)	Increase tensile and burst strength Decrease tear resistance	[235]
Chitosan and cellulose nanofibers	Paper recycling (decolorization)	Remove water-based inks	[236]
Microparticules with chitosan and bentonite	Paper reinforcement	Chitosan is a good dry strength additive	[237]
Chitosan as additive	Papermaking (aging stability of paper)	Increase tensile strength. Decrease the hydrophilicity of paper	[238]
Chitosan with zeolite as filler	Papermaking	Improve the mechanical properties of paper	
Chitosan as additive	Paper reinforcement (Kenaf paper (*Hibiscus cannabinus*))	Give a good mechanical and dry strength properties	[239]
Graphene ink from the exfoliation of graphite in pullulan, chitosan, and alginate	For strain-sensitive paper	Paper-based strain sensor, the chitosan-graphene has the best resistivity value and demonstrates the highest sensitivity towards strain	[240]

The chitosan can also be used as amino-functionalized structures for CO_2_ capture. Many industrial processes could reduce their emissions using these systems. Furthermore, there are many other options where chitosan can be used to reduce the greenhouse gas emissions [241]. Table 10 displays the chitosan-based materials used for gas capture.

**Table 10:** Chitosan-based materials used for gas capture.

Adsorbate	Adsorbent	Effects	References
Carbon dioxide	Composite with chitosan and clay	Adsorption capacity of 344.98 mg/g	[242]
Carbon dioxide	Arginine-containing chitosan-graphene oxide aerogels	CO_2_ gas adsorption was equal to 24.15 wt% (5.48 mmol g^−1^)	[243]
Palladium (II) and platinum (IV)	Cross-linked chitosan	340.3 mg/g and 203.9 mg/g for Pd and Pt, respectively (adsorption capacity)	[244]
Carbon dioxide (separation)	Membrane with carboxymethyl chitosan and carbon nanotubes	Good CO_2_ selectivity and permeability	[245]
Carbon dioxide	Acetic acid-mediated chitosan	368 mg/g adsorption capacity Good CO_2_ Selectivity	[246]
Carbon dioxide	Chitosan as a porosity agent	280.5 mg/g adsorption capacity	[247]
Formaldehyde gas	Chitosan crosslinked with metal-organic framework (MOF-199)@aminated graphene oxide aerogel	197.89 mg/g adsorption capacity	[187]
Carbon dioxide	Chitosan-grafted multi-walled carbon nanotubes	CO_2_ uptake capacity was found to be significantly higher (1.92 ccg^−1^)	[248]

## 4. Future Perspectives

It is expected that chitosan uses will increase replacing other traditional materials due to its interesting properties and functionalities, but also due to it being abundant, it can be extracted using green chemistry and easily treated as waste. For these reasons, chitosan is considered a rich renewable resource where some of its shortcomings associated with solubility, mechanical properties, and porosity are being addressed due to the potential of this source.

This article shows some of the most prominent fields where chitosan is an interesting alternative to other conventional materials, but its properties will be reflected soon in other many fields due to its versatility and properties. Some of the most promising applications could be associated with specific areas such as medicine, food packaging, or biotechnology, among others.

There is a lot of room to grow in terms of the production of chitosan, the current goal of which is clearly focused on the removal of hazardous solvents and reducing the energy consumption. On the other hand, chitosan can contribute to sustainability in terms of recycling and waste management due to its degradability.

## 5. Conclusions

Chitosan shows an interesting range of properties which make it very useful for sustainable development due to it being abundant, biodegradable, biocompatible, and versatile. The production of chitosan is improving in terms of green chemistry, due to the hazardous chemicals being replaced by eutectic solvents, lower energy consumption has been achieved, and circularity can be applied to secondary processes. The use of chitosan in films for food packaging shows better properties than traditional films composed of polyethylene. The edible food packing with enhanced antimicrobial activity can be developed using chitosan. Numerous blends of chitosan have been developed with various essential oils and extracts which are excellent antibacterial and antifungal agents. On the other hand, the chitosan provides interesting and multiple features for a sustainable agriculture, such as a protection for the plant and increasing the production. Finally, the chitosan can contribute to green chemistry in multiple processes such as the paper industry or the treatment of wastewater, reducing the impact and contributing to the circularity of industrial processes. The chitosan-based composites, hydrogels, and membranes can be used for the remediation of diversified pollutants including dyes, antibiotics, phenols, metal ions, etc. Thus, being a second abundant biopolymer in nature, chitosan can be a potential sustainable future material.
